# Patents as Early Indicators of Technology and Investment Trends: Analyzing the Microbiome Space as a Case Study

**DOI:** 10.3389/fbioe.2018.00084

**Published:** 2018-06-20

**Authors:** Manuel Fankhauser, Christian Moser, Theodor Nyfeler

**Affiliations:** ^1^Seerave Foundation, St. Helier, Jersey; ^2^Arven Partners LLC, Bern, Switzerland; ^3^Swiss Federal Institute of Intellectual Property (IPI/IGE), Bern, Switzerland

**Keywords:** patent analysis, big data, investment trends, microbiome, cancer

## Abstract

The human microbiome is the collective of microbes living in symbiosis on and within humans. Modulating its composition and function has become an attractive means for the prevention and treatment of a variety of diseases including cancer. Since the initiation of the human microbiome project in 2007, the number of academic publications and active patent families around the microbiome has grown exponentially. Screening patent databases can be useful for the early detection and the tracking of new technology trends. However, it is not sufficient to assess portfolio sizes because emerging players with small but high-quality patent portfolios will be missed within the noise of large but low-quality portfolio owners. Here we used the consolidated database and software tool PatentSight to benchmark patent portfolios, and to analyze key patent owners and innovators in the microbiome space. Our study shows how in-depth patent analyses combining qualitative and quantitative parameters can identify actionable early indicators of technology and investment trends from large patent datasets.

## Assessing the quality of patent portfolios

Screening patent databases can be useful for the early detection and the tracking of new technology trends. What makes such screening particularly useful is the fact that emerging trends rarely receive mainstream attention early on, since they are mainly driven by academic research and startups under the radar. This opens interesting possibilities for early-stage investors, foundations, VCs and innovation scouts who need to identify institutions or individuals driving a given technology space. However, it is not sufficient to assess portfolio sizes because emerging players with small but high-quality patent portfolios will be missed within the noise of large but low-quality portfolio owners.

Here we used PatentSight, a consolidated database and software tool to benchmark patent portfolios, and to analyze key patent owners and innovators in the microbiome space. Key measures of this analysis are quality parameters assigned to each patent family, such as the Technology Relevance^TM^ (TR, measured by the number of citations received), Market Coverage^TM^ (MC, measured by the protected market size), and the Competitive Impact^TM^ (CI, the product of TR and MC). The Patent Asset Index^TM^ (PAI) represents the cumulated business value of a patent portfolio, calculated as the sum of the CIs of the patents included therein (Ernst and Omland, 2011).

## Exponential activity in the microbiome space

**Figure 1:**
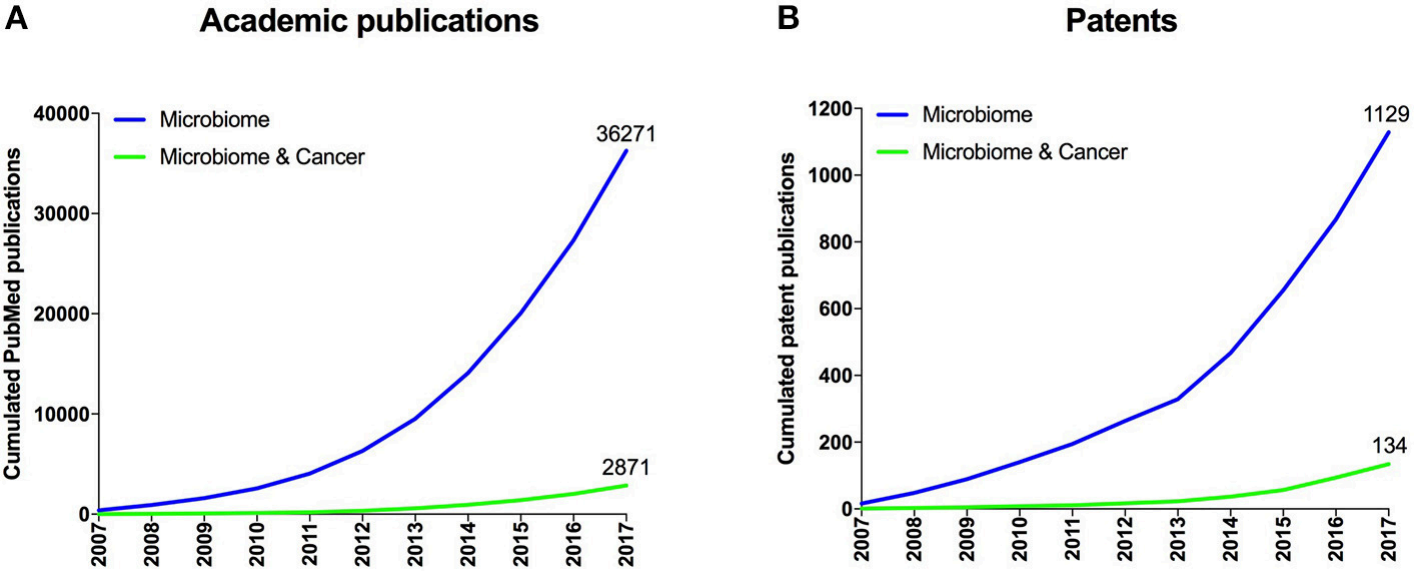
Overall published academic research publications and patent families. **(A)** Cumulated number of academic publications retrieved from the PubMed database related to microbiome research and to cancer research within the microbiome space. **(B)** Cumulated number of patent families related to microbiome research and to cancer within the microbiome space from consolidated data extracted from multiple patent databases.

The human microbiome is the collective of microbes including bacteria, viruses and fungi living in symbiosis on and within humans. Given that the human microbiome has also been implicated in a variety of diseases including cancer, modulating its composition and function has become an attractive means for the prevention and treatment of those diseases (Lynch et al., 2016; Young, 2017). Accordingly, the number of academic publications and active patent families around the microbiome has grown exponentially (Figure 1) since the initiation of the human microbiome project in 2007 (Turnbaugh et al., 2007). However, even though an astonishing amount of scientific data has been generated over the last few years, their translation into products for patients has so far been difficult. While crude fecal microbiome transplantations have been widely used in a variety of indications, the first standardized microbiome therapeutics (such as defined bacterial consortia, microbiome-modulating drugs or prebiotics, and bacteriophages) are currently under clinical evaluation. A better understanding of the established and emerging microbiome stakeholders including research institutions, inventors, and companies should enable a more systematic approach to developing collaborations and partnerships that aim at advancing safe and standardized microbiome diagnostics and therapeutics to clinics.

## Academia and startups capture some of the most valuable portfolios

**Figure 2:**
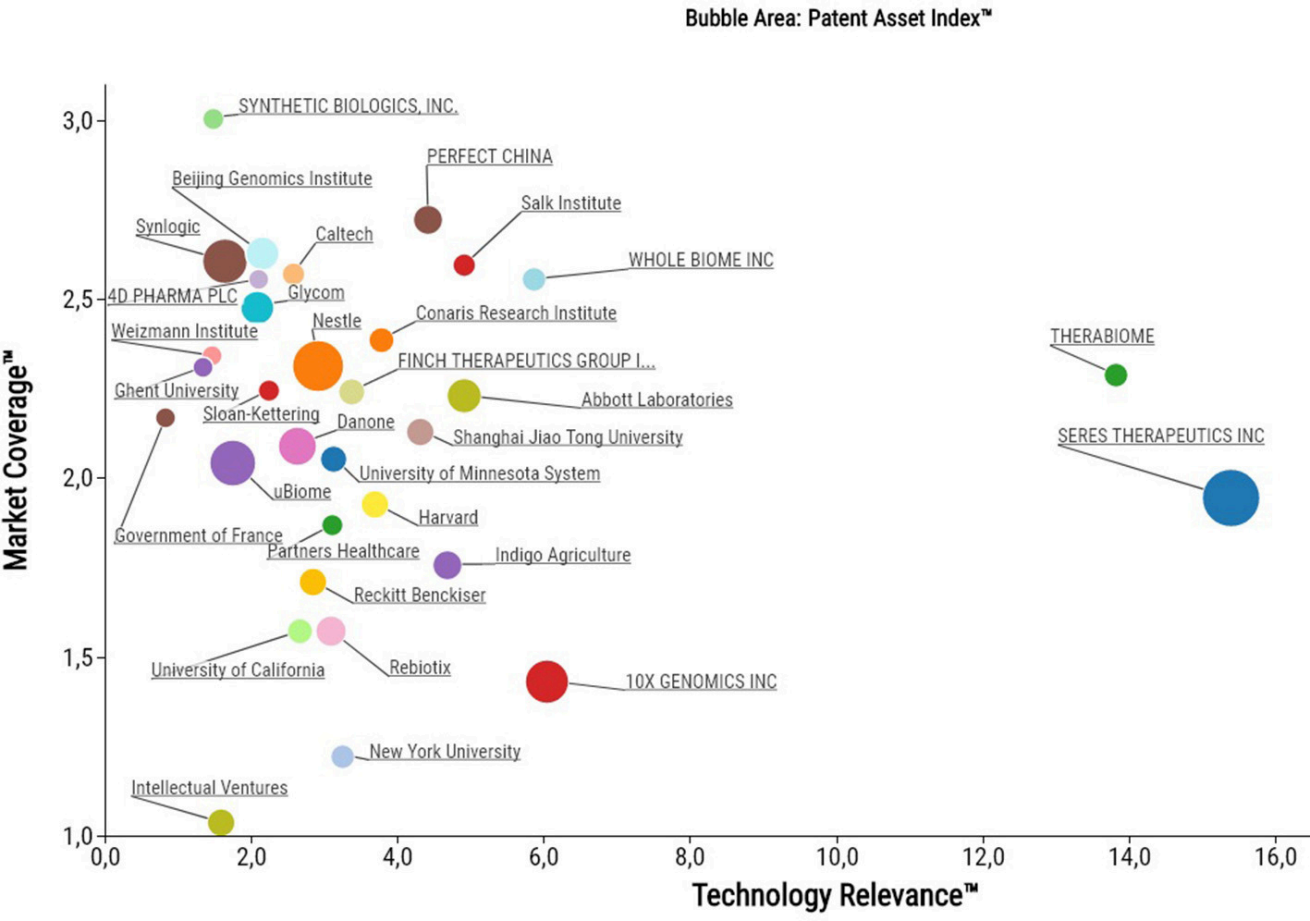
Bubble plot of the top 32 microbiome patent owners as assessed and plotted with PatentSight (reporting date: 31.12.2017).

So who are the driving individuals, academic institutions or companies in this emerging space of the life sciences? While others have previously taken biased approaches to characterize the microbiome patent space in terms of classifying patent families into certain commercial areas or disease indications (Consultancy, 2016; Sun et al., 2016; Sabatelli et al., 2017), we focused on an unbiased approach to assess quality parameters. Our in-depth patent analysis using PatentSight revealed that a diverse mix of top patent owners from large industry, startups and academia are holding some of the key patent assets (Figure 2). This includes multinational food companies (e.g., Nestlé and Danone), startup companies that recently did an IPO (e.g., Synlogic Therapeutics in 2017 and Seres Therapeutics in 2015), an agriculture technology unicorn (Indigo), but also multiple startups that have so far been largely under the radar (e.g., Finch Therapeutics, Whole Biome, Therabiome).

While there is a homogenous distribution across Market Coverage, Seres Therapeutics and Therabiome stand out in terms of Technology Relevance. Despite of their small portfolio sizes, these companies own some of the cornerstone patent families relevant to the entire microbiome field. Their high technology relevance results from being frequently cited by patent examiners as state-of-the-art.

## Quality indicators reveal complementary insights to portfolio size

**Figure 3:**
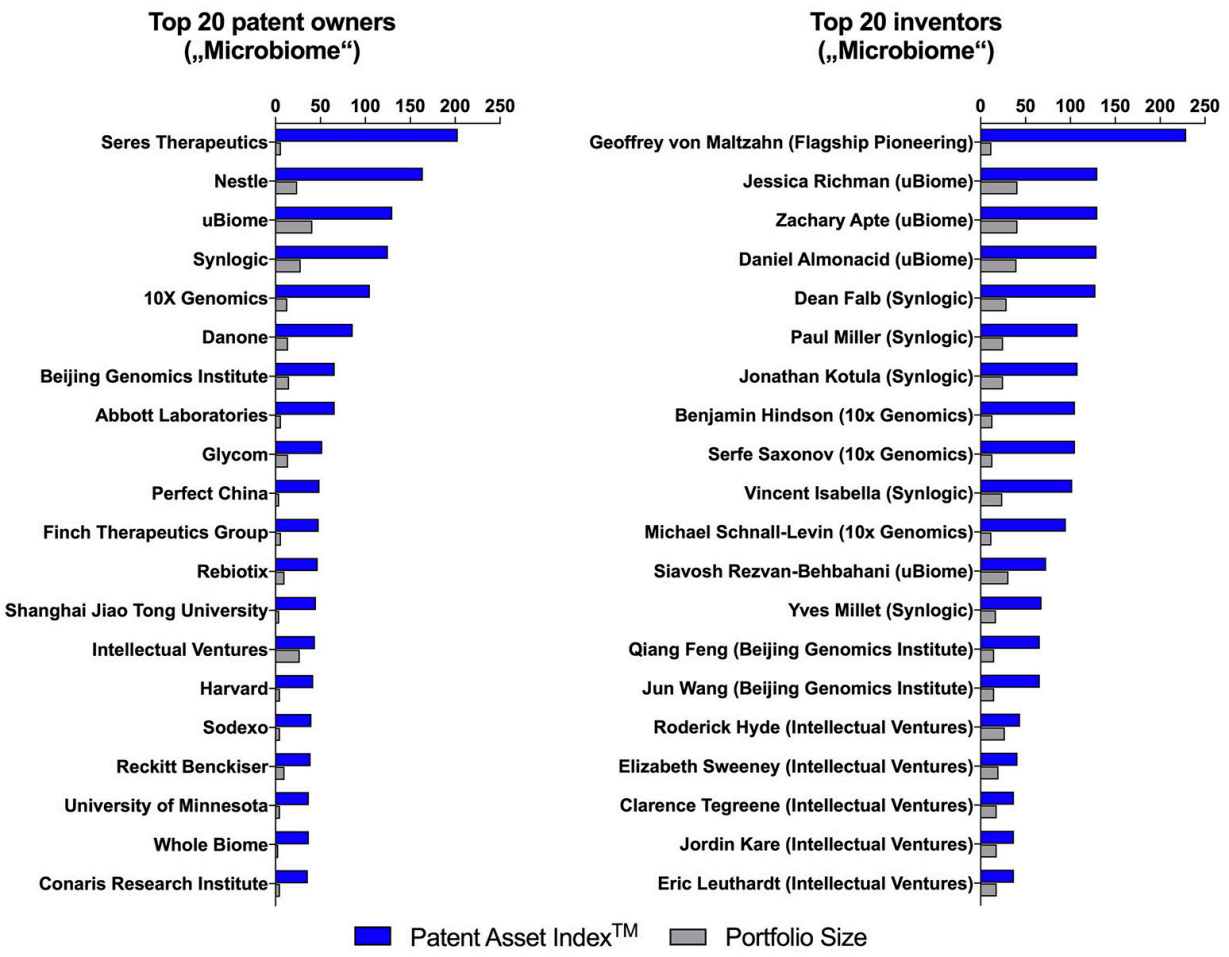
Patent Asset Index^TM^ and portfolio size of the top 20 microbiome patent owners and inventors with their current affiliation.

The quality of patent portfolios as determined by the Patent Asset Index^TM^ (the sum of all Competitive Impacts of an entire portfolio) does not correlate with portfolio size and provides an independent more sophisticated method to rank the top 20 microbiome patent owners (Figure 3, left side) and inventors (Figure 3, right side). This approach reveals top microbiome players with focused patent portfolios, which in a classic portfolio size analyses can be overshadowed by large but low-quality patent portfolios. This is for example illustrated when comparing Seres Therapeutics, a startup with a small but high-quality portfolio, with Nestlé, a multinational with a large but low-quality portfolio. One of the leading patent inventors identified using our approach, Geoffrey von Maltzahn, is a partner at the venture capital firm Flagship Pioneering and co-inventor on patent families of multiple microbiome startups including Seres Therapeutics, Kaleido Biosciences and Indigo Agriculture.

**Figure 4:**
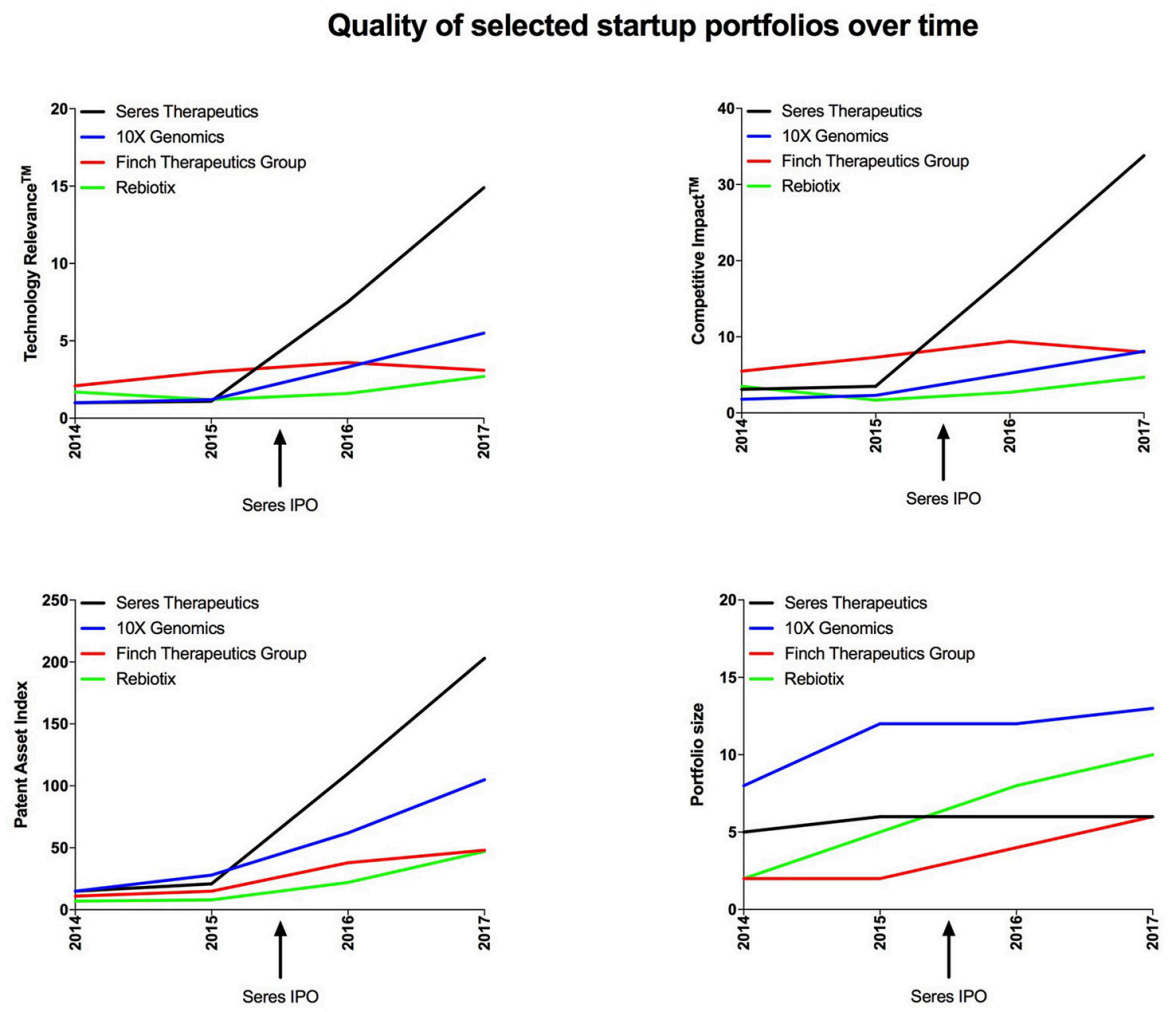
Portfolio quality and size of the 4 startup companies with the highest Technology Relevance^TM^ by 2017 plotted over time.

In addition to cross-sectional analysis, plotting quality indicators over time can provide insights into the business strategy of startup companies, such as preparing an IPO, an M&A, or expanding to new markets ahead of time (Figure 4). The IPO of Seres Therapeutics in June 2015 could have only been predicted by detecting subtle changes that started appearing a few months before, emphasizing the need to constantly track qualitative indicators. The acquisition of Rebiotix by Ferring Pharmaceuticals earlier this year was possibly hinted at by the subtle increases in Technology Relevance and Patent Asset Index observed since 2016 (Figure 4, left two graphs). Interestingly, the merger of Finch Therapeutics Group with OpenBiome in 2017 may have increased their patent portfolio size, but compromised the overall quality (Figure 4, bottom two graphs). Finally, being a post-Series C venture-backed startup that has steadily increased patent portfolio quality since 2014, it is conceivable that 10X Genomics will undergo an exit within the coming months. In the future, machine learning algorithms may be applied in order to predict such upcoming events with more and more accuracy.

## Identifying emerging trends within the microbiome space

**Figure 5:**
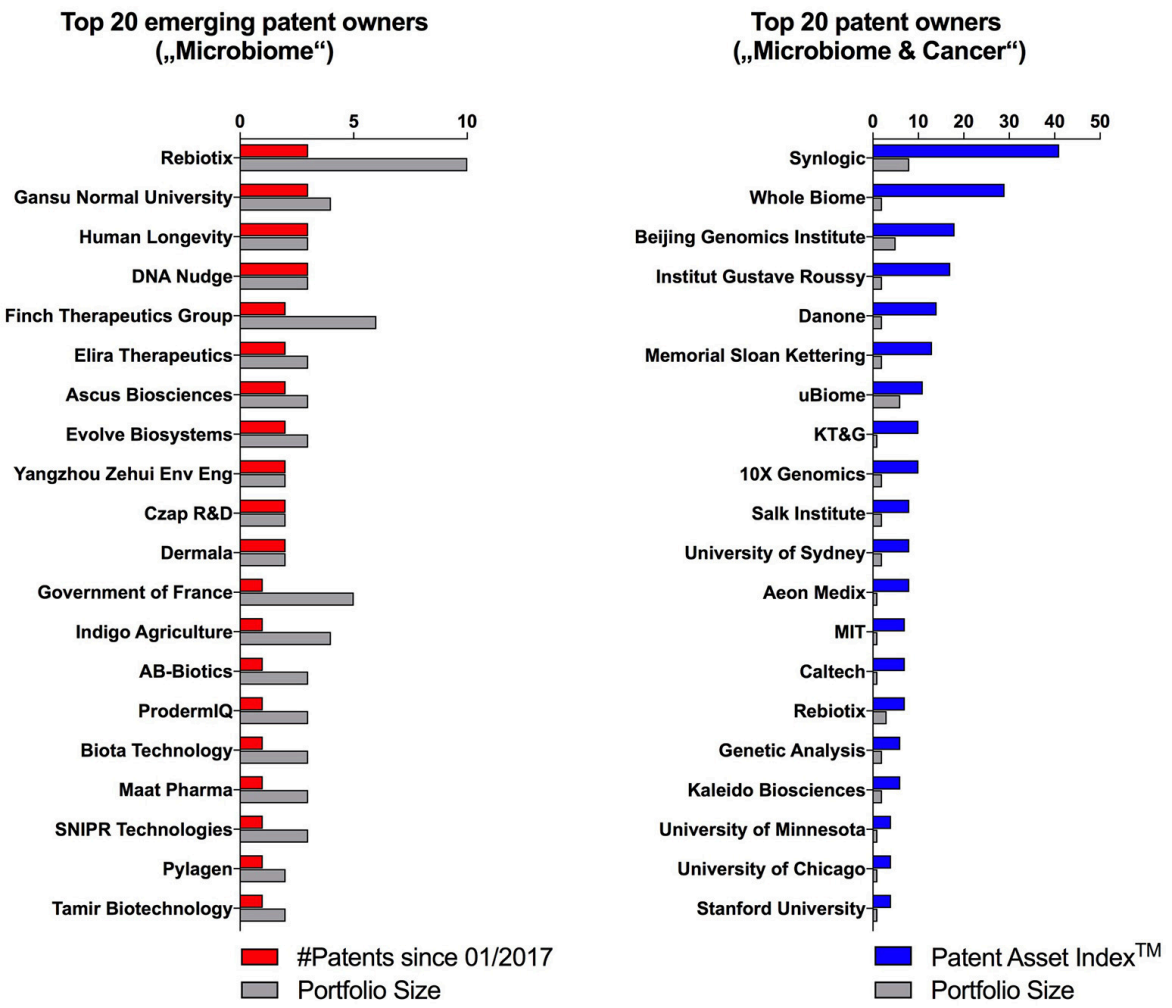
Emerging patent owners with equal or less than 10 patents ranked according to published patents since January 2017, and the Patent Asset Index^TM^ and portfolio size of the top 20 microbiome patent owners looking at using the microbiome for diagnostic or therapeutic approaches in cancer.

The early detection of emerging trends within the microbiome patent space may help to identify both novel scientific developments as well as new exciting early-stage ventures. To identify the latter, we filtered patent owners with portfolio sizes of equal or less than 10 patent families and ranked them according to the number of patents published since January 2017 (Figure 5, left side). This approach highlighted entities which have entered the microbiome space very recently, and thus, may not yet have received widespread attention. One such example is SNIPR Technologies LTD, a private company incorporated in the UK in 2015 with no publicly available data or website. Based on its patent descriptions, this startup seems to apply CRISPR technologies in order to alter human microbial populations. Another example is Human Longevity Inc. founded by genomics pioneer Craig Venter. Even though this company has been in the public focus for a while, is has been mainly known for its ambition to build health insights based on large human genomic data sets. The fact that they published their first three microbiome patent families within only one year (2017) may reflect a pivot in company strategy.

To explore a novel scientific trend, we filtered patent owners that use microbiome approaches for the diagnosis or treatment of cancer, which we identified to be an emerging disease focus within the overall microbiome space (Figure 1). Interestingly, this analysis brought forward a new set of entities (Figure 5, right side) including some universities (e.g., Institut Gustave Roussy and Memorial Sloan Kettering) and startups (e.g., Kaleido Biosciences) that did not appear in the prior, unfiltered analysis. Cancer as an emerging disease focus of microbiome diagnostics and therapeutics was further validated by the recent appearance of high impact academic publications covering this topic (Gopalakrishnan et al., 2017; Routy et al., 2017; Matson et al., 2018).

Taken together, the presented in-depth patent analyses adding quality parameters to quantity can identify actionable early indicators of technology and investment trends from large patent datasets. The resulting insights are suitable for further evaluation by more targeted approaches applied in more traditional due diligence processes.

## Limitations

The case study presented here contains a number of limitations, due to the search methodology and the availability of information, and most importantly, because of the dynamics of a rapidly evolving technology field.

First, the analysis relies on a set of patent families selected by two keyword concepts, notably without reviewing the entire set of several thousand individual documents. This implies that irrelevant documents may be included. More importantly, documents relevant to the topic may be missing in the analysis set due to the shortcomings of the search queries. Optimizing the search queries to maximize the precision and the completeness of the resulting analysis set represents in our view the most decisive factor for the quality of the study results. Accordingly, a large part of the time dedicated to this study was invested in this task, which is a laborious, iterative process requiring in-depth understanding of the technical field as well as professional patent searching skills.

Second, the patent databases are updated on a regular basis for changes of ownership but licensing deals are not included in the records and thus invisible. A significant proportion of the microbiome patent families is owned by public research organizations, as illustrated in Figure 2. Universities frequently license their inventions to third parties, while maintaining the ownership of their patents. As a result, the respective licensees, start-ups as well as established companies remain invisible until they file patent applications on their own within the technology field of interest.

Third, the rapidly growing number of patent families in the microbiome field with widely dispersed ownerships result in a highly dynamic patent landscape. The deeper analyses shown in Figures 2–5 represent spotlights on the status at the end of 2017, and the picture is likely to change in the future. Therefore, regular updates of the entire analysis are necessary in order to track changes and new trends, and most importantly, to identify players maintaining a prominent profile over an extended period of time. Finally, the lag time of 18 months between initial filing of a patent and its publication causes an unavoidable blind spot for patent searches. This lag time affects also PatentSight's quality parameter Technology Relevance^TM^ (TR), which is based on forward citations. Citations require prior publication, availability on databases, and recognition by researchers. Thus, citations come into play only after an additional delay. Therefore, the TR and its related quality parameters are less reliable for very recently published patent families.

## Methods

The patent documents used in this analysis were retrieved from various patent databases, including Epodoc, Derwent World Patent Index (DWPI), PatBase^1^, and a collection of over 50 full-text patent databases in English, German, and French available via EPOQUEnet^2^ The keyword search concepts for microbiome (microbiome, microbiota, commensal microflora) and for cancer (cancer, tumor, carcinoma, neoplasm, malignancy, proliferative disease, proliferative disorder) included synonyms and spelling variations in English and German. The search concepts were further refined based on relevant and irrelevant documents retrieved by the initial searches. Furthermore, various index fields available in the databases were explored as targets for the keyword search. Finally, the mature keyword concepts were applied to searches in the various patent databases mentioned above, targeting the index fields title, abstract, and claims where available, but excluding documents featuring the keywords in the full-text only.

**Figure 6:**
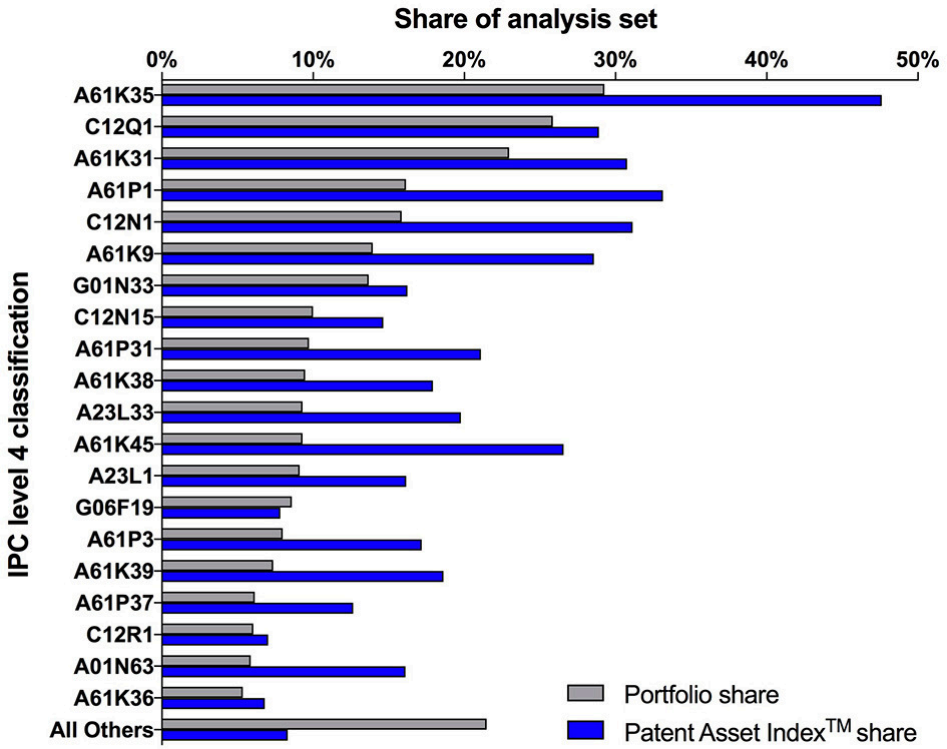
International patent classification (IPC) statistics of the analysis set of 1,142 patent families, including the inactive families. The bars show the shares of the top 20 IPC level 4 classifications with regard to number of patent families (Portfolio share, gray bars) and with regard to their cumulated value (Patent Asset Index™ share, blue bars). Detailed descriptions of the IPC classifications are available via the website of the World International Patent Origanization (WIPO). (World Intellectual Property Organization website, IPC classifications: Available online at: http://www.wipo.int/classifications/ipc/en/).

Searching relevant documents via patent classification systems, such as the international patent classification (IPC) or the cooperative patent classification system (CPC), was explored as an option but ultimately abandoned. When applied as stand-alone search concepts, patent classifications retrieved document sets with an unacceptably high proportion of irrelevant documents. Conversely, the combination of patent classifications with the keyword concepts limited the sets unnecessarily and excluded relevant documents. Patent classifications can be extremely useful for searches in established technology fields, but are much less productive to capture an emerging technology with a broad variety of applications, as it is the case for the microbiome field. Not surprisingly, the patent classification statistics of the final, consolidated analysis set showed a wide distribution of the patent families over three sections, with the top ten IPC level 4 classifications covering less than 80% of the entire set (Figure 6).

In order to estimate the proportion of relevant and irrelevant documents within a set retrieved by a given query, a subset of documents was generated by random sampling. Depending on the size of the set retrieved, 20–50 documents were sampled and reviewed individually in order to determine their relevance. Using this approach, the keyword search concepts were iteratively refined in several rounds to achieve an optimal balance between precision and comprehensiveness. Based on this quality control procedure by random sampling, around 95% of the over 5,000 retrieved documents within the final set used for analysis are expected to be relevant to the topic.

n a last step, the patent documents collected in the various databases were all transferred to the patent analysis tool PatentSight^3^ Therein, the documents were grouped into patent families, and duplicates were eliminated. A patent family represents a single invention and encompasses all related documents—patent applications as well as granted patents worldwide—referring back to the same first patent application, the priority application. This consolidation leads to a significant reduction in numbers within the analysis set, in the current study from over 5,000 documents retrieved down to 1,142 patent families, thereof 956 active patent families as of 15 December 2017. The distinction between active and inactive patent families relies on their legal status. For a given time point, only those patent families qualify as active which comprise at least one pending application or one patent in force. Expired, abandoned, or rejected patent families are kept on record, but by definition, inactive patent families have a Market Coverage™ value of zero, and thus, no impact on the quality parameters Competitive Impact™ or Patent Asset Index™.

## Author contributions

TN, MF, and CM designed the analysis strategy. MF and CM performed the analysis, curated and consolidated the data set and wrote the manuscript with input and revisions from TN. MF and CM contributed equally to this work.

### Conflict of interest statement

MF is employed by Arven Partners LLC. The remaining authors declare that the research was conducted in the absence of any commercial or financial relationships that could be construed as a potential conflict of interest.

## Footnotes

^1^PatBase: commercial patent search and analysis tool: Available online at: https://minesoft.com/our-products/patbase/

^2^EPOQUEnet is a professional patent search system available to EPO examiners and national patent offices to query over 170 databases. Available online at: https://www.epo.org/service-support/glossary.html#e.

^3^PatentSight: commercial patent search and analysis tool including proprietary patent quality parameters: Available online at: https://www.patentsight.com/en-us/.
